# Environmental Co-Exposure to Potassium Perchlorate and Cd Caused Toxicity and Thyroid Endocrine Disruption in Zebrafish Embryos and Larvae (*Danio rerio*)

**DOI:** 10.3390/toxics10040198

**Published:** 2022-04-18

**Authors:** Davide Di Paola, Sabrina Natale, Carmelo Iaria, Rosalia Crupi, Salvatore Cuzzocrea, Nunziacarla Spanò, Enrico Gugliandolo, Alessio Filippo Peritore

**Affiliations:** 1 Department of Chemical, Biological, Pharmaceutical and Environmental Science, University of Messina, 98166 Messina, Italy; davide.dipaola@unime.it (D.D.P.); sabrina.natale@unime.it (S.N.); carmelo.iaria@unime.it (C.I.); aperitore@unime.it (A.F.P.); 2 Department of Veterinary Science, University of Messina, 98166 Messina, Italy; rcrupi@unime.it (R.C.); egugliandolo@unime.it (E.G.); 3 Department of Pharmacological and Physiological Science, School of Medicine, Saint Louis University, Saint Louis, MO 63103, USA

**Keywords:** environment contaminant, heavy metals, oxidative stress, thyroid function

## Abstract

The increasing pollution of aquatic habitats with anthropogenic compounds has led to various test strategies to detect hazardous chemicals. However, information on the effects of pollutants on the thyroid system in fish, which is essential for growth, development, and parts of reproduction, is still scarce. Modified early life-stage tests were carried out with zebrafish exposed to the known thyroid inhibitor potassium perchlorate (0.1, 1, 1.5, 2, 2.5, and 5 mM) to identify adverse effects on embryo development. The endogenous antioxidant defense mechanism is one of the key functions of the thyroid gland; in this regard, we examined the co-exposure to potassium perchlorate (KClO_4_), which could disrupt thyroid function, with cadmium (Cd), a known pro-oxidant compound. Zebrafish embryos were exposed to control KClO_4_ 1 mM and Cd 0.5 μM for 96 h after fertilization (hpf) individually and in combination. The morphological alteration, body length, and messenger RNA (mRNA) expression related to thyroid function and oxidative stress, thyroid hormone levels, and malondialdehyde were measured. Significant down-regulation of mRNAs related to thyroid function (thyroid hormone receptor-alpha (THRα), thyroid hormone receptor-beta (THRβ), haematopoietically expressed homeobox (hhex)) and decreased thyroxin (T4) levels were observed after co-exposure to KClO_4_ and Cd, but this was not observed in the individually treated groups. These results suggest that co-exposure to KClO_4_ and Cd could affect antioxidant defense mechanisms and potentially normally increase Cd toxicity on mRNA expression, altering the thyroid functions important in zebrafish embryonic developmental stages.

## 1. Introduction

In fish, thyroid hormones play a key role in development, growth, metabolism, and survival in the early stages of life, particularly in the larval stage. Possible disruption of thyroid function during sensitive stages of fish development could lead to health impairment and even growth retardation [1]. Thyroid hormone activity is exceptionally important during early development in amphibians and fish because it is responsible for the completion of metamorphosis [2,3,4]. The importance of thyroid hormones in this phase of development in fish is exceptionally evident in flatfish, which are dependent on thyroid hormones to metamorphose into asymmetric juveniles [2,3,4]. The first thyroid follicle in zebrafish differs around 55 h after fertilization (hpf), while at 72 hpf, thyroxine (T4) production begins [5]. In the adult zebrafish, the first follicle corresponds to the anterior follicle, which plays a key role in histopathological evaluations because it can vary in size depending on whether conditions are pathological or non-pathological [6]. Moreover, it is well known that during the first three days of zebrafish growth (sufficient for the development of most organs), the embryo depends on the maternal THs stored in the yolk sac because the thyroid gland is not yet formed [7]. Several chemicals present in the environment have been reported to disrupt thyroid action in fish [8]. Among the various chemicals in the environment, perchlorate pollution has been widely documented, especially in the United States. Potassium perchlorate (KClO_4_) can be used as a solid oxidant for rocket propulsion, and it was the original source of a fraction of the contamination. In fact, perchlorate concentrations from 8 ng/mL to 3.7 mg/mL have been reported in the groundwater and surface water in several western states [9]. Between 2001 and 2005, the U.S. EPA conducted nationwide perchlorate sampling, detecting perchlorate levels of 4 μg/L or higher in approximately 4.1% of the public water systems examined, and in some of these, it was as high as 15 μg/L or more [10]. Data from the Department of Defense between 1997 and 2009 revealed perchlorate concentrations ranging from less than 1 μg/L to 2.6 g/L [10]. The perchlorate concentrations used in the present study cover the range reported by these studies to allow conclusions on the environmental status. Perchlorate is known to affect thyroid function through competitive inhibition of the sodium/iodide symporter, which is responsible for iodide uptake into thyrocytes. In fact, perchlorate blocks iodide uptake into the thyroid by affecting the sodium/iodide symporter of the thyroid follicle on the thyroid epithelium, inhibiting TH synthesis [11,12].

In addition, it has been reported that thyroid hormones may also regulate antioxidant levels in fish, suggesting that altered thyroid homeostasis may affect not only growth but also the response to oxidative stress [13]. As a result, substances that influence thyroid gland function in fish may likewise increase pro-oxidant chemical toxicity. Heavy metals in different environments arise from natural sources, such as erosion, volcanic activity, and forest fires, raising great concern regarding aquatic environments, reaching through contaminated sediments, wastewater, and oil spills [14]. In aquatic organisms, cadmium (Cd) has been shown to cause oxidative stress [15]; DNA damage, direct suppression of enzyme activity, and lipid oxidation are all possible outcomes [16,17]. Cd has been employed in a variety of industrial and commercial activities for decades [18]. Several studies have shown that Cd exposure from 6 hpf onward can affect the hatching rate of zebrafish larvae as well as swimming ability and circadian cycle in a concentration-dependent manner [19,20]. Furthermore, it has also been shown that Cd exposure in a time-dependent manner can cause damage to the zebrafish brain, leading to neurological toxicity [19].

We expected that preexposure to a sublethal amount of KClO_4_ would impair thyroid function, increasing the toxicity of Cd, a pro-oxidant chemical, in zebrafish at early life stages. Various molecular-, biochemical-, and organism-level endpoints were identified after fertilized zebrafish eggs were subjected to various combinations of KClO_4_ and Cd exposure for 96 hpf. In the present study, we used two widespread environmental contaminants, potassium perchlorate (KClO_4_) and cadmium (Cd), as model chemicals and investigated the potential consequences of combined exposure to both chemicals during the early life stages of zebrafish (*Danio rerio*).

## 2. Materials and Methods

### 2.1. Zebrafish Maintenance and Embryo Collection

Wild-type (WT) mature zebrafish with an age of 6 months were used for the production of embryos. The University of Messina Center of Experimental Fish Pathology (Centro di Ittiopatologia Sperimentale della Sicilia, CISS, Messina, Italy) supplied zebrafish maintenance and fertilized egg collection. The fish were fed dry and live food twice a day at an amount of 3% of their body weight (BW). Mature females and males were mated in a 2:1 ratio for successful reproduction. The eggs were collected the next day in a chamber at 28 °C and bleached, and non-fertilized eggs were discarded. For the experiments, only embryos that had reached the blastula stage were employed. The FET (Fish Embryo Toxicity) test was carried out in accordance with OECD guidelines [21] and ISO 15088.

### 2.2. Survival Rate, Hatching Rate, and Morphology Score

Healthy embryos were implanted in 24-well culture plates at 4 h post-fertilization (hpf) (1 embryo in 2 mL solution/well). Zebrafish embryos were exposed to potassium perchlorate (KClO_4_) and cadmium chloride (CdCl_2_) (all treatment solutions were made in reconstituted water) for 24–96 hpf to measure the toxic effects over a continuing observation period. First, preliminary experiments consisting of varying concentrations of KClO_4_ were conducted to determine the concentration–response curve and after being tested in association with non-toxic concentrations of Cd. Fertilized eggs were transferred into 24-well plates with test solutions and incubated at 28 °C with a 14:10 h day/night light regime. The embryo medium was composed of 15 mM NaCl, 0.5 mM KCl, 1 mM CaCl_2_, 1 mM MgSO_4_, 0,15 mM KH_2_PO_4_, 0.05 mM Na_2_HPO_4_, and 0.7 mM NaHCO_3_ at a pH 7.3. Briefly, embryos were exposed to water only (blank control); KClO_4_ at nominal concentrations of 0.5, 1, 1.5, 2, 2.5, and 5 mM; and Cd at 0.5 μM (3 replicates; 20 eggs in each replicate; 3 independent experiments). The KClO_4_ and Cd solutions were changed daily, and the overall survival rate and developmental abnormalities of the embryos and larvae were monitored and photo-recorded at 24, 48, 72, and 96 hpf [22]. During the period of exposure to the substances, the embryos/larvae were observed every 24 h for abnormalities in development, survival, and hatching rate, as well as morphological changes [23]. Morphologic score evaluation occurred at 96 hpf using 9 endpoints as previously described [24]; briefly, nine endpoints, namely body shape, somites, notochord, tail, fins, heart, face, brain, and pharyngeal arches/jaws, were examined to evaluate the phenotypes of the zebrafish, and eight larval specimens per group were used for scoring. Photographs of the embryos were obtained under a stereomicroscope (Leica M0205C, multifocal). Fresh larval specimens were killed with an overdose of the anesthetic MS-222 (tricaine methane sulfonate) at a dose greater than 0.6 g/L before sampling for various analyses.

### 2.3. Thyroid Hormone Measurement

Levels of triiodothyronine (T3) and thyroxine (T4) were determined as previously described [25]. Briefly, at 96 hpf, the larvae were sacrificed and homogenized in 1 mL enzyme-linked immunosorbent assay buffer using a homogenizer, vortexed for 10 min, and centrifuged at 5000× *g* for 10 min at 4 °C. The supernatant was collected and used for the analysis. T3 and T4 were measured by an enzyme-linked immunosorbent assay following the manufacturer’s recommendations (USCN Life).

### 2.4. Gene Expression Analysis

Total RNA from zebrafish larvae was extracted, reverse-transcribed, and amplified according to the instructions of the manufacturer of the kits and as described previously [26]. Table 1 shows the detailed information on the primers as previously reported [27,28]. β-actin was used as an internal control for normalizing relative expression levels between samples [29,30,31]. Data analysis was performed using the 2^−∆∆Ct^ method, and the results are expressed as fold changes.

### 2.5. Lipid Peroxidation

The assessment of lipid peroxidation was performed by a malondialdehyde (MDA) assay described in [32]. Each sample included 30 larvae (96 hpf) that were pooled in an Eppendorf tube, and for measurement, the pooled larvae were homogenized using extraction buffer (with 87.575 g sucrose, 200 mL glycerol, 700 mL 100 mM phosphate buffer, and 0.5 μL phenylmethanesulphonylfluoride). The homogenate was centrifuged at 25,525× *g* for 15 min, and then supernatant was used for measurement. Malondialdehyde (MDA) levels were measured as thiobarbituric acid-reactive substances, which are products of reactive oxygen species-induced lipid peroxidation.

### 2.6. Statistical Evaluation

For multiple comparisons, a two-way/one-way ANOVA was used, followed by a Bonferroni post hoc test. The Kolmogorov–Smirnov test (*p <* 0.05) was used to check for normal distributions, and the data are reported as means and standard deviation (SD) (alpha value of 0.05). Graphpad Prism 8 was used for statistical analysis. Non-significant differences in the mean are indicated by bars with the same letter, whereas statistically significant differences in the mean are indicated by bars with different letters.

## 3. Results

### 3.1. Survival, Hatching Rate, Morphology, and Thyroid Hormone Levels

**Figure 1:**
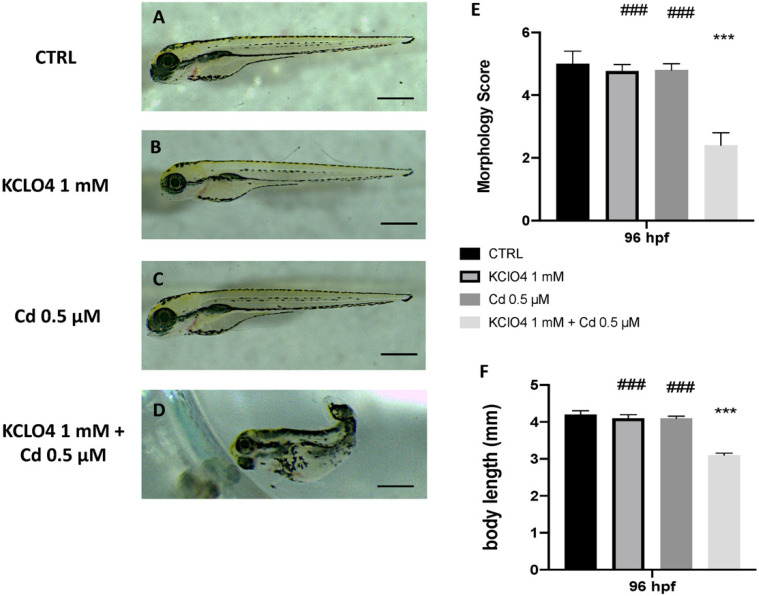
Effects of KClO4 and Cd single and co-exposure on morphological changes in zebrafish larvae at 96 hpf. CTRL (**A**), KClO4 (**B**), Cd (**C**), KClO4 + Cd (**D**). Morphology Score (**F**) and body length (**E**) of zebrafish larvae treated. *** *p* < 0.001 versus CTRL; ^###^ *p* < 0.001 versus KClO_4_ + Cd.

To determine the most appropriate concentrations and time points for the following experiments, KClO_4_ at 0.5, 1, 1.5, 2, 2.5, and 5 mM was applied to observe the morphology, survival, and hatching rate of embryos/larvae (Table 2). As presented in Figure 1B, KClO_4_ 2 at 2.5 and 5 mM induced zebrafish embryo mortality at 24 hpf of around 100%. KClO_4_ at 0.5 and 1 mM did not show mortality at 24 hpf, while at 96 hpf, mortality was shown to be less than 3%. However, KClO_4_ at 1.5 mM showed a mortality of 51% at 96 hpf, and no effect was seen at 24 hpf. CTRL was normal; hatching began at 48 to 72 hpf. Hatching was also normal at KClO_4_ concentrations of 0.5 and 1 mM, while no hatching was seen for the concentrations of 2, 2.5, and 5, at which the embryos died after 24 h post exposure. Furthermore, a decrease in the hatching rate was seen for KClO_4_ 1.5 mM compared with the CTRL group at 72 and 96 hpf (Table 2). Compared with the CTRL group, exposure to KClO_4_ at 0.5 and 1 mM had no effect on zebrafish morphology until 96 hpf. Phenotypic defections at time points up to 96 hpf were noted only in the KClO_4_ 1.5 mM group (the higher concentrations died at 24 hpf), which showed a spinal axis curve at 96 hpf (Figure S1). In addition, thyroid hormone levels were assessed at 96 hpf for different exposures of KClO_4_, and only the highest concentration showed alterations in expression compared with CTRL, with an increase in T3 and a decrease in T4 levels.

Subsequently, we evaluated the toxic effect of Cd exposure at different concentrations (0.05, 0.5, and 1 μM) on the morphology, survival, hatching, and thyroid hormone levels (Table 2). Decreases in the survival rate and hatching rate were observed for the Cd 1 μM exposure, as well as altered levels of thyroid hormone. No differences were found in the group exposed to Cd at 0.05 and 0.5 μM compared with CTRL in terms of malformations, survival, hatching, and thyroid hormone levels.

### 3.2. Toxic Effect of Combined Exposure to KClO_4_ and Cd on Malformation and Body Lenght

**Figure 2:**
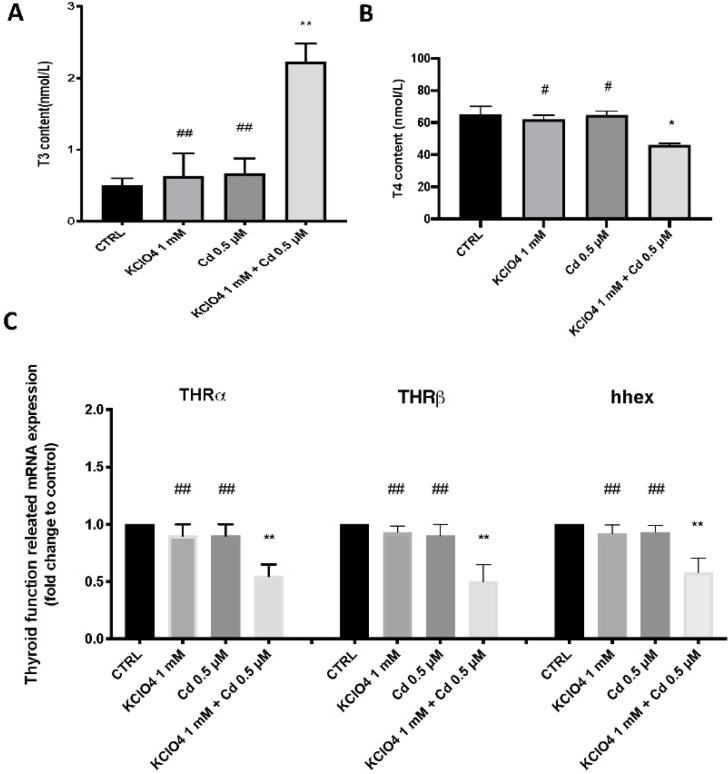
Whole-body concentration of (**A**) triiodothyronine (T3); and (**B**) thyroxine(T4) in zebrafish larvae exposed to KClO_4_ and Cd alone and in combination for 96 hpf. (**C**) The expression of THRα, THRβ, and hhex mRNA was quantified using qPCR. Values are means ± SD of three independent experiment data. The expression levels of mRNA are represented as the fold change from the CTRL group. * *p* < 0.05, ** *p* < 0.01; ^##^ *p* < 0.01 versus KClO_4_ + Cd, ^#^ *p* < 0.05 versus KClO_4_ + Cd.

Toxic effects of KClO_4_ at a 1 mM concentration and Cd at 0.5 μM were seen on malformation at 96 hpf. At this point, we used the highest non-toxic concentration of both KClO_4_ and Cd, selected from our previous studies [33], to highlight any synergic toxic effect. The morphology score of the single-exposure KClO_4_ and Cd group showed no significant change compared with CTRL. Moreover, when embryos were co-exposed to both KClO_4_ and Cd, developmental defects were seen (Figure 2). Abnormalities, pericardial edema, and scoliosis were found in the KClO_4_ and Cd co-exposure group at 96 hpf (Figure 1). The body lengths of the larvae were measured at 96 hpf to assess the degree of development (Figure 1). The KClO_4_ and Cd group larvae had considerably shorter body lengths at 96 hpf, indicating that the combination of modest doses of KClO_4_ and heavy metals dramatically slowed larval growth. There were no variations in body length between the groups exposed to KClO_4_ or Cd alone.

### 3.3. Thyroid Function after Single Exposure and Co-Exposure to KClO_4_ and Cd

Treatment with KClO_4_ and Cd co-exposure significantly increased T3 levels compared with the control group and the single exposure (Figure 2). By contrast, the T4 concentration was decreased in the larvae exposed to co-exposure compared with the CTRL and single exposure groups (Figure 2B). Exposure to KClO_4_ and Cd at early life stages in zebrafish led to significant differences in thyroid-related function genes (THRa, THRb, and hhex), which were significantly down-regulated in the co-exposure group but not in the single exposure groups at 96 hpf (Figure 2).

### 3.4. Effect of KClO_4_ and Cd Antioxidant Pathway and Lipid Peroxidation

**Figure 3:**
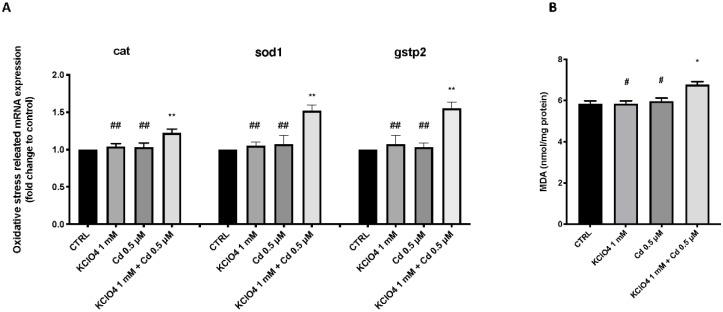
Effects of KClO_4_ and Cd single exposure and co-exposure on the mRNA levels of the stress oxidative pathway (cat, sod, and gstp2) (**A**) in larval zebrafish; malondialdehyde (MDA) levels (**B**). Values are means ± SEM of three independent experiments’ data; * at *p* < 0.05 against CTRL; ** at *p* < 0.01 against CTRL; ^#^ *p* < 0.05 versus KClO_4_ + Cd; ^##^ *p* < 0.01 versus KClO_4_ + Cd.

Figure 3 shows the changes in the expression of antioxidant-related genes in zebrafish larvae after exposure to KClO_4_ and Cd alone and in combination. The KClO_4_ and Cd co-exposure group exhibited significantly upregulated expression levels of cat, sod1, and gstp2 compared with the CTRL group. However, there was no significant change in the expression level of antioxidant-related genes in the KClO_4_ and Cd single-exposure groups (Figure 3).

## 4. Discussion

The current study demonstrated that co-exposure to KClO_4_ and Cd in the early life stages of zebrafish could increase the toxicity of the individual non-toxic contaminants. The toxic action of co-exposure could occur through alteration of thyroid functions that are related to responses to oxidative stress. The first objective of the study was to identify an appropriate non-toxic concentration within a KClO_4_ range to then co-expose zebrafish to Cd to evaluate the potential synergistic effect. We demonstrated that KClO_4_ induced developmental toxicity in zebrafish embryos, specifically delayed hatching and morphological abnormalities. At concentrations of 2, 2.5, and 5 mM, all embryos showed mortality as early as 24 hpf, whereas lower doses did not significantly induce mortality. The concentration that showed almost 50% mortality of larvae at 96 hpf was 1.5 mM. The 1.5 mM KClO_4_ exposure not only showed clear signs of toxicity, reducing survival, but also decreased the hatching rates at 72 and 96 hpf compared with the lowest concentrations analyzed. Hatching is a critical time in zebrafish embryogenesis; therefore, the decrease in the hatching rate was induced by functional and structural disturbances during embryonic development [34,35]. In addition, the suppression of embryogenesis or inhibition of mitosis [36] or the inability of embryonic larvae to open the chorion [37] possibly contributed to the developmental delay. In addition, exposure to 1.5 mM KClO_4_ led to embryonic teratogenesis, which was characterized by spinal curvature in 96 hpf larvae. It has been reported that mean dissolved Cd concentrations in rivers are strongly impacted by mining activities; in the Riou Mort in France, the concentration is 26 μg/L [38]. Because fish have a poor metabolic rate and clearance of heavy metals, the effects of Cd may be persistent in zebrafish [39]. Critical pathways involved in developmental toxicity are oxidative stress and inflammation [40,41]. Regarding contaminant-induced oxidative stress, two main mechanisms are involved: the reduction of the cellular antioxidant defenses and ROS overproduction [42,43]. ROS are crucial in the oxidative stress pathway, promoting the progression of the process, and combine with the action of CAT, GSH, and SOD to create an imbalance in antioxidant defenses. [44,45]. SOD is an antioxidant enzyme responsible for the removal of ROS and prevents lipid peroxidation, another process involved in the defense mechanism of our bodies [46,47]. A member of the GST Pi family, gstp2, also participates in the process of ROS removal by interaction with glutathione [48], while CAT is an important enzyme that converts H_2_O_2_ to water and oxygen [49]. The mRNA levels of sod1, cat, and gstp2 increased after co-exposure to KClO4 and Cd, showing an over-stimulation of the antioxidant defenses that does not occur in the case of single exposure to these two contaminants.

Following early exposure to contaminants, a reduction in body length during zebrafish embryonic development was reported, accompanied by T4 and T3 level alterations [50,51]. THs make several contributions to scale formation and pigmentation during the transition from late larva to young zebrafish [1]. T4 and T3 are the major forms generated by the thyroid gland and play key roles in growth and development.

Previous studies have shown that the effect of the presence of contaminants on T3 and T4 expression is not always equal. The thyroid endocrine toxicity of cadmium chloride in zebrafish embryos and larvae has been reported. A previous study showed that T4 and T3 levels measured in the larvae of parents exposed to cadmium chloride were significantly decreased, consistent with the results of the thyroid hormone levels detected after exposure to other environmental toxicants [52]. Our data are in line with previous statements about a decrease in T4 levels as well as thyroid receptors, but not about T3 levels. Indeed, reductions in total and free serum T4 levels and an increase in serum T3 have been found simultaneously following exposure to environmental contaminants in zebrafish larvae [32]. Since serum T3 is 80% the product of the conversion of T4 by 5′-deiodination in peripheral tissues, and the remaining 20% is secreted by the thyroid [53], one could speculate that this difference in expression was due to increased conversion of T3 from T4 with potentially reduced T4 production. In our research, both T3 and T4 levels were markedly altered after co-exposure to KClO_4_ and Cd, but not after the single exposure. Thus, zebrafish tend to be affected by exogenous TH and thyroid inhibitors during the transition phase [1].

During the embryonic and early larval stages of zebrafish, the mRNA profiles of thyroid hormone receptor-alpha (THRα) and thyroid hormone receptor-beta (THRβ) are developmentally controlled, and their expressions are known to be drastically raised until the yolk-sac larval stage [54]. Thyroid hormone activities are mediated by these nuclear receptors; hence, variations in THRα and THRβ mRNA expression are significant [55]. The fact that larvae co-exposed to KClO_4_ and Cd were more susceptible to single exposure emphasizes the importance of synergic toxicity during the early stages of a zebrafish’s life when its thyroid gland is developing into a functional one. The changes in THRα and THRβ mRNA expression are important because thyroid hormone actions are mediated by these nuclear receptors. Moreover, haematopoietically expressed homeobox (hhex) is important for thyroid follicle differentiation and formation and can be used as a marker gene in zebrafish thyroid development [56]. Decreased hhex mRNA expression after co-exposure to KClO_4_ and Cd suggests a potential reduction in thyroid gland development early in a zebrafish’s life at 96 hpf, which is not seen in the presence of only one of these two contaminants.

## 5. Conclusions

Our data demonstrated that exposure to sublethal concentrations of KClO_4_ can increase the susceptibility of zebrafish larvae to pro-oxidants, such as Cd. Although the concentrations used in our study are slightly higher than those reported to date in the environment, the results support what has been seen in other work, i.e., that thyroid hormones play a role in regulating antioxidant levels in fish [13]. Further studies are needed to clarify the ecotoxicological risks of long-term exposure to these environmental contaminants. Moreover, since there are several lines of scientific evidence on the potential cross-link between endocrine disruptors and the antioxidant system, further research efforts should be made to understand the mechanisms of their interactions and, consequently, the risks of the release of these substances into the water.

## Figures and Tables

**Table 1:** Primers for real-time PCR.

Gene	Accession Number	Primer Orientation	Nucleotide Sequence
b-actin	NM_131031	forward	5′-CTTCCAGCAGATGTGGATCA-3′
		reverse	5′-GCCATTTAAGGTGGCAACA-3′
hhex	NM_130934.1	forward	5′-TGTGGTCTCCGTTCATCCAG-3′
		reverse	5′-TTTGACCTGTCTCTCGCTGA-3′
THRa	NM_131396.1	forward	5′-CAATGTACCATTTCGCGTTG-3′
		reverse	5′-GCTCCTGC TCTGTGTTTTCC-3′
THRb	NM_131340.1	forward	5′-TGGGAGATGATACGGGTTGT-3′
		reverse	5′-ATAGGTGCCGATCCAATGTC-3′
sod1	NM_131294.1	forward	5′-GGCCAACCGATAGTGTTAGA-3′
		reverse	5′-CCAGCGTTGCCAGTTTTTAG-3′
cat	NM_130912.2	forward	5′-AGGGCAACTGGGATCTTACA-3′
		reverse	5′-TTTATGGGACCAGACCTTGG-3′
gstp2	NM_001020513	forward	5′-CACAGACCTCGCTTTTCACAC-3′
		reverse	5′-GAGAGAAGCCTCACAGTCGT-3′

**Table 2:** Cd effects on zebrafish larvae endpoints and thyroid hormone levels. * *p* < 0.05 versus CTRL; ** *p* < 0.01 versus CTRL; *** *p* < 0.001 versus CTRL.

	Survival		Hatching		Morphology	Thyroid Hormone	
	72 h	96 h	72 h	96 h	96 h	T3	T4
CTRL	100 ± 0	100 ± 0	100 ± 0	100 ± 0	ND	1.49 ± 0.005	78.33 ± 4.40
KClO_4_ 0.5 mM	100 ± 0.57	98 ± 2	99.22 ± 0.57	100 ± 0.57	ND	1.51 ± 0.008	76 ± 2.64
KClO_4_ 1 mM	100 ± 0.57	97.33 ± 3.78	99 ± 0.10	100 ± 0.57	ND	1.53 ± 0.006	75.67± 2.40
KClO_4_ 1.5 mM	71 ± 1 ***	51.67 ± 3.05 ***	80.67 ± 2.08 ***	93 ± 3 ***	Scoliosis	1.98 ± 0.07 ***	61 ± 3.05 **
KClO_4_ 2 mM	0	0	0	0	ND	//	//
KClO_4_ 2.5 mM	0	0	0	0	ND	//	//
KClO_4_ 5 mM	0	0	0	0	ND	//	//
Cd 0.05 μM	100 ± 0	99 ± 0.57	100 ± 0	100 ± 0	ND	1.51 ± 0.015	77 ± 4.16
Cd 0.5 μM	99.67 ± 0.33	98.67 ± 0.66	98.67 ± 2.02	100 ± 0	ND	1.55 ± 0.003	76.67 ± 3.33
Cd 1 μM	99.33 ± 0.33	91.67 ± 0.88 *	96.33 ± 2.02 *	96.33 ± 2.02 *	ND	1.63 ± 0.038 *	64.33 ± 3.92 *

## Supplementary Materials

The following are available online at https://www.mdpi.com/article/10.3390/toxics10040198/s1, Figure S1: The morphological defections in zebrafish caused by KClO4 different concentrations exposure. SC—Scoliosis. Images were taken from the lateral view under a dissecting microscope (magnification 25). Scale bar, 500 mm.
