# Supplementary material for: Environmental Co-Exposure to Potassium Perchlorate and Cd Caused Toxicity and Thyroid Endocrine Disruption in Zebrafish Embryos and Larvae (Danio rerio)
Source: Toxics. 2022 Apr 18;10(4):198. doi: 10.3390/toxics10040198 (PMC9030446; doi:10.3390/toxics10040198)
Supplement: Supplementary file 1 [file toxics-10-00198-s001.zip › toxics-1658617-supplementary.pdf]

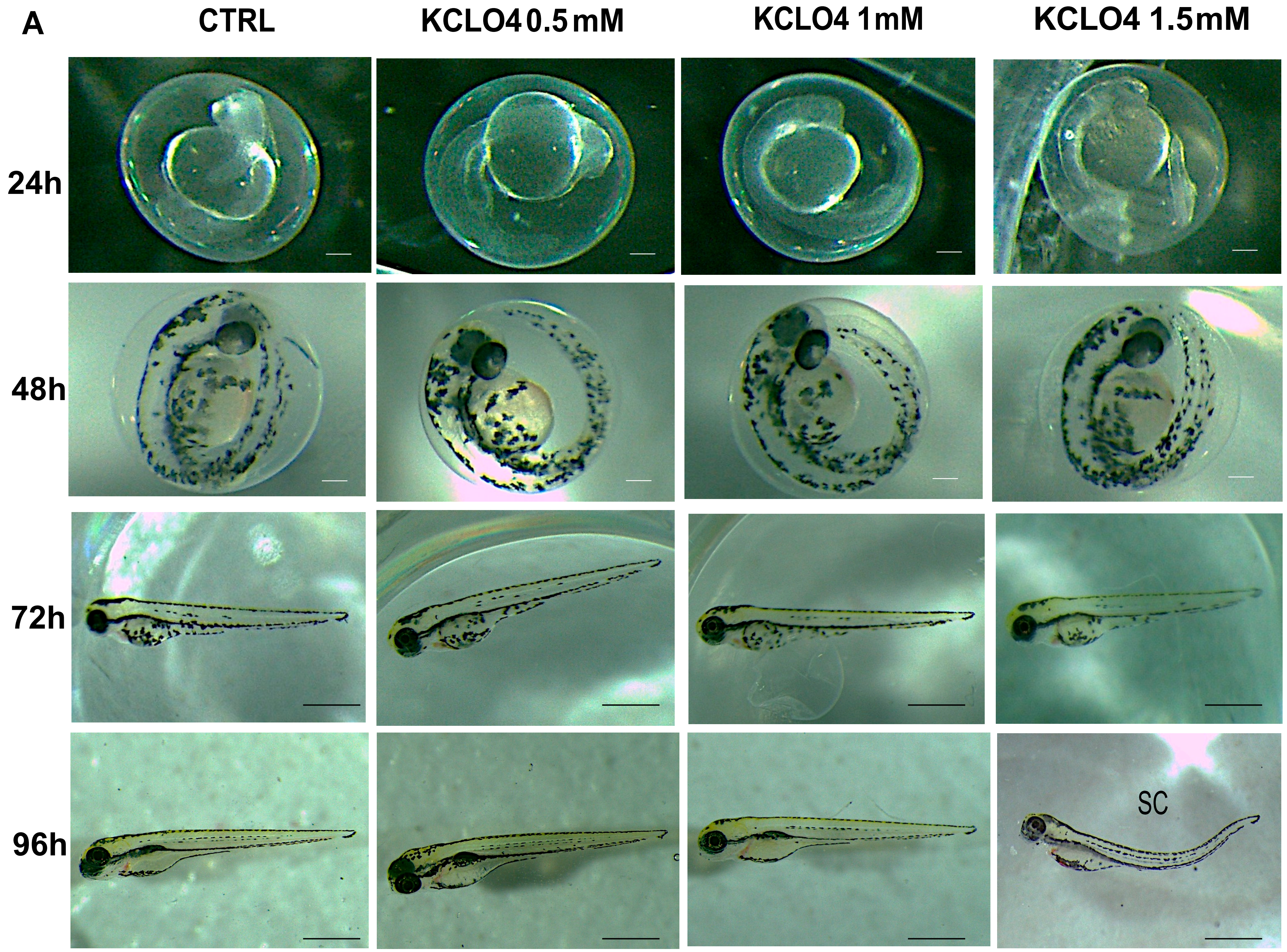

Figure S1. The morphological defections in zebrafish caused by $KClO_4$ different concentrations exposure. SC – Scoliosis. Images were taken from the lateral view under a dissecting microscope (magnification 25). Scale bar, 500 mm.
